# Evaluation of Serum Ferritin Levels and Their Correlation With Clinical Outcomes in Dengue Virus Infection in a Population of Older Adults

**DOI:** 10.1111/tmi.70113

**Published:** 2026-03-06

**Authors:** Sacha Orberg Temer, Thaís Guimarães, Augusto Yamaguti, João Silva de Mendonça

**Affiliations:** ^1^ Infectious Diseases Department Hospital Do Servidor Público Estadual de São Paulo São Paulo Brazil

**Keywords:** biomarker, ferritin, older adults, severe dengue

## Abstract

**Objectives:**

Prior studies have associated elevated ferritin levels with worse dengue outcomes but data from older adults remain limited. The aim of this study is to evaluate the serum ferritin levels and their correlation with disease severity in a population of older adults.

**Methods:**

We included 284 patients aged 60 years or older with a diagnosis of dengue attended during a high dengue fever outbreak. We analysed demographic variables, the presence of comorbidities, hospitalisation, death and correlated the serum ferritin level with three categories of dengue. Patients' ferritin levels were recorded during the febrile (P1), critical (P2) and convalescence (P3) phases.

**Results:**

Of the 284 patients, 59.1% of whom were women, with a mean age of 72 years, ranging from 60 to 99 years. Of these, 228 (80.2%) had some clinical comorbidity. 106 (37.3%) of patients were classified as dengue without warning signs; 122 (43%) as dengue with warning signs and 56 (19.7%) as severe dengue. During P1, 68 (23.9%) serum ferritin levels were collected with an average result of 511 ng/mL; during P2, 143 (50%) serum ferritin levels were collected with an average result of 1697 ng/mL, and during P3, there were 141 (49.6%) collections with an average level of 949 ng/mL. 111/284 (39%) of patients requiring hospitalisation and 9/284 (3.1%) died, all of them diagnosed with severe dengue. Our study found higher levels of ferritinemia in cases of severe dengue and in the critical (723 × 1420 ng/mL; *p* = 0.006) and convalescent (554 × 1261 ng/mL; *p* = 0.03) phases, with statistically significant differences.

**Conclusions:**

Our study found higher levels of ferritinemia in cases of severe dengue and in the critical and convalescent phases, with statistically significant differences. Age over 60 may be associated with the development of severe dengue, and hyperferritinemia may represent a biomarker of dengue severity.

## 1 Introduction

Approximately a decade ago, it was estimated that around 400 million dengue virus (DENV) infections occurred globally each year, with about one‐quarter presenting as symptomatic disease, resulting in nearly 10,000 deaths annually [1, 2]. More recently, a mathematical model combining meteorological and socioeconomic data projected an increase in the global population at risk of dengue in the coming decades [3]. This model predicted that tropical and subtropical regions would be particularly affected due to longer warm seasons, which favour the survival of the mosquito vector.

In 2024, Brazil experienced its worst dengue epidemic in recorded history, with unprecedented numbers of dengue‐related cases and deaths [4]. A similar trend was observed across the Americas, with approximately five times the average number of cases reported over the past 5 years, reflecting increased incidence in all subregions [5].

Symptomatic dengue cases range in severity from mild, self‐limiting illness—comprising the vast majority—to severe disease and death. Early identification of patients at higher risk of progressing to severe dengue is critical, especially in scenarios of potential healthcare system overload, which is common in low and middle‐income countries. In 2009, the World Health Organization introduced a classification for dengue with warning signs, recommending hospitalisation for at least 48 h due to the higher risk of severe disease [6]. However, a subsequent prospective multicentre study raised concerns about unnecessary hospital admissions using this classification [7].

The importance of identifying objective prognostic markers associated with the pathophysiology of severe dengue has also been emphasised [8, 9]. Although the mechanisms underlying severe dengue are not fully understood, they are known to involve the “cytokine storm” phenomenon [10]. As in other conditions linked to cytokine storms, such as hemophagocytic syndrome and COVID‐19, hyperferritinemia has emerged as a potential prognostic biomarker for severe outcomes in dengue [11, 12, 13].

The objective of the present study was to evaluate the serum ferritin levels and their correlation with disease severity in a population of older adults in a dengue‐endemic region of Latin America.

## 2 Methods

### 2.1 Study Population

A population of older adult patients (≥ 60 years old) attended at the Hospital do Servidor Público Estadual de São Paulo located at São Paulo, Brazil, between March and June 2024 during a high dengue fever outbreak and that met the inclusion criteria: clinical syndrome compatible with dengue (fever associated with two or more symptoms of headache, myalgia, arthralgia, nausea and vomiting, rash, retro‐orbital pain, petechial, leucopenia and positive capillary fragility test) and a laboratory confirmation of active dengue due to NS1+ or positive IgM in a rapid test. Serum ferritin was collected between the first and fourteenth days of symptoms at the discretion of the attending physician.

Patients with co‐infections at the time of dengue diagnosis were excluded. We consider co‐infection if the patient had another infectious disease during this same hospitalisation, that is, viral infections such as COVID‐19, Influenza or bacterial infections with bacteremia caused by *S. aureus* or another etiological agent.

### 2.2 Data Collection

Data were extracted from the patients' electronic medical records retrospectively and summarised in a Microsoft Excel worksheet.

We analysed demographic variables, and the presence of specific comorbidities was also registered, such as chronic kidney disease, hepatopathy, diabetes mellitus, active solid cancer or haematological malignant neoplasm, heart disease and immunosuppression. Immunosuppression was considered present in patients living with HIV/AIDS, solid organ transplant recipients, those with haematological neoplasms, chronic kidney disease, chronic liver disease, active solid organ cancer or patients undergoing therapeutic immunosuppression.

Ferritin sampling when collected was divided into three periods: the febrile phase (P1, from the first to the third day of illness); critical phase (P2, from the fourth to the seventh day of illness) and convalescence phase (P3, from the eighth to the fourteenth day of illness), according to the typical expected evolution of dengue and the ferritin values found in a previous study [11].

The method used for quantifying serum ferritin in our laboratory is immunochromatography (Ferritin, Beckman Coulter Inc., USA) and the levels considered normal are 30 to 300 ng/mL for men and 10 to 200 ng/mL for women. This method has a maximum limit of 7500 ng/mL.

### 2.3 Outcomes of Interest

We classified the progression of dengue into three categories, according to WHO criteria [6]: (1) dengue without warning signs (DwoWS), which is for patients who had fever and at least two of the following symptoms: headache, retro‐orbital pain, myalgia, arthralgia, rash or prostration; (2) dengue with warning signs (DwWS) that was defined if the patient had intense abdominal pain (spontaneous or during physical examination), persistent vomiting, postural hypotension or lipothymia, hepatomegaly (> 2 cm below the costal margin), hemoconcentration, mucosal bleeding and/or lethargy or irritability, and: (3) severe dengue (SD) which is characterised by manifestations such as haemorrhages, impairment of the circulatory system, and/or involvement of organs.

Patients who presented warning signs and later developed severe dengue were classified as severe dengue.

We compare serum ferritin levels in these three disease categories.

We also evaluated hospitalisation and hospital mortality.

### 2.4 Statistical Analysis

Analysis of the qualitative variables was based on the determination of association using Pearson's chi‐square test or Fisher's Exact Test when the assumption to apply the χ^2^ was not satisfied. For the analysis of the differences in the medians or means of quantitative variables, Kruskal‐Wallis's method was used. Potential factors related to disease severity were compared through univariate analysis, and all factors identified as significant by this analysis were subjected to multivariate analysis. The independent variables were expressed through their odds ratios, and their respective 95% confidence intervals were estimated. All reported significance probabilities were two‐sided and calculated considering a significance level of 0.05% or 5.0%. The statistical analysis was performed using SPSS software version 25.

### 2.5 Ethics

This study was approved by the Ethics Committee for Research of our hospital under the identifying number 81023124.9.0000.5463.

## 3 Results

Our study included 284 patients, 59.1% of whom were women, with a mean age of 72 years, ranging from 60 to 99 years. Of these, 228 (80.2%) had some clinical comorbidity. 106 (37.3%) of patients were classified as DwoWS; 122 (43%) as DwWS and 56 (19.7%) as severe dengue.

During Phase 1, 68 (23.9%) serum ferritin levels were collected with an average result of 511 ng/mL; during Phase 2, 143 (50%) serum ferritin levels were collected with an average result of 1697 ng/mL, and during Phase 3, there were 141 (49.6%) collections with an average level of 949 ng/mL.

111 of 284 (39%) of patients requiring hospitalisation and 9 of 284 (3.1%) died, all of them diagnosed with severe dengue.

The characteristics of the patients included are summarised on Table 1.

**TABLE 1:** Characteristics of the patients included in the study (*N* = 284).

Variable	DwoWS *N* = 106	DwWS *N* = 122	SD *N* = 56	*p*	OR (95% CI)
Gender female	72 (67.9%)	67 (54.9%)	29 (51.7%)	0.06	
Age (mean in years)	72	71	76	0.0001	1.07 (1.03–1.10)
**Serum ferritin levels (median in ng/mL)**					
P1 (febrile phase; from the first to the third day of illness) *N* = 68	294	301	244	0.80	
P2 (critical phase; from the fourth to the seventh day of illness) *N* = 143	431	1356	1420	< 0.0001	*
P3 (convalescence; from the eighth to the fourteenth day of illness) *N* = 141	315	678	1261	0.0012	*
Comorbidities	81 (76.4%)	96 (78.6%)	51 (91%)	0.07	
Cardiopathy	14 (13.2%)	19 (15.5%)	13 (23.2%)	0.15	
Diabetes mellitus	28 (26.4%)	20 (16.3%)	22 (39.2%)	0.002	2.48 (1.33–4.60)
Renal chronic disease	0 (0)	2 (1.6%)	6 (10.7%)	NA	
Cancer	4 (3.7%)	7 (5.7%)	5 (8.9%)	0.39	
Hepatopathy	0 (0)	4 (3.2%)	2 (3.5%)	0.08	
Immunosuppression	5 (4.7%)	15 (12.2%)	19 (33.9%)	0.78	
Hospitalisation	7 (6.6%)	54 (44.2%)	50 (89.2%)	0.20	
Time until hospitalisation (mean in days)	3.7	4.7	4.4	0.67	
Length of stay (mean in days)	4	5	11	0.0018	3.46 (2.34–6.55)
Death	0 (0)	0 (0)	9 (16%)	NA	

Abbreviation: NA, not applicable.

* OR is shown in Table 2.

Cases of dengue without warning signs and dengue with warning signs were grouped and considered non‐severe. So, we compared them with cases of severe dengue. Serum ferritin levels in cases of non‐severe and severe dengue are described in Table 2.

**TABLE 2:** Comparison of serum ferritin levels between non‐severe and severe dengue cases.

Serum ferritin levels (median in ng/mL)	Non‐severe dengue (*N* = 228)	Severe dengue (*N* = 56)	*p*	OR (95% CI)
P1 (febrile phase; from the first to the third day of illness)	294	244	0.63	NA
P2 (critical phase; from the fourth to the seventh day of illness)	723	1420	0.006	1.0002 (1.0001–1.0004)
P3 (convalescence; from the eighth to the fourteenth day of illness)	554	1261	0.03	1.0003 (0.999–1.0006)

Abbreviation: NA, not applicable.

## 4 Discussion

We analysed a cohort of predominantly female patients of older adults with comorbidities. Although it was not possible to differentiate primary from secondary dengue virus infection, 62.7% of cases progressed with warning signs or to severe cases. Severe dengue was more prevalent in older individuals with comorbidities and diabetes, and they required longer hospital stays. The mortality rate of 3.1% also occurred only in severe cases.

Our study also found higher levels of ferritinemia in cases of severe dengue and in the critical and convalescent phases, with statistically significant differences.

Ferritin, an acute‐phase reactant and iron‐storage protein, has emerged as a promising biomarker associated with dengue severity. Ferritin levels increase significantly during systemic inflammation and immune activation. In dengue infection, elevated ferritin is thought to reflect excessive immune response, including macrophage activation and cytokine release. Several studies have demonstrated that patients with severe dengue exhibit markedly higher serum ferritin levels compared to those with non‐severe disease [10, 11, 12, 13]. Hyperferritinemia has been associated with complications such as shock, bleeding, liver injury and multiple organ dysfunction [14].

Previous studies demonstrate that hyperferritinemia can differentiate dengue from other febrile diseases, which may be a result of dengue virus tropism for the hepatocytes and cells of the mononuclear lineage which are known to produce ferritin in response to inflammatory stimuli, such as IL‐1β, IL‐6, TNF‐α [9, 15, 16].

Multiple studies have reported the association of serum ferritin levels with the severity of dengue disease [7, 9]. Assays for the estimation of serum ferritin are easy to perform and comparatively less expensive, and serum ferritin levels may be considered as a prognostic marker to predict severe dengue [9].

A systematic review and meta‐analysis published in 2023 analysed 18 studies examining serum ferritin levels in dengue cases in the context of disease severity. This meta‐analysis suggests that serum ferritin may be a marker of disease severity [12]. However, the studies analysed were almost entirely conducted in India. Our study is one of the few conducted in Latin America, where there is high endemicity of dengue and outbreak periods, and it was carried out in a specific population of older adults who have more comorbidities and greater exposure to the virus over their lifetime.

In our cohort, mean serum ferritin levels were significantly higher both in the second period of disease (P2) and in the third period (P3) for severe dengue. It should be noted that a prospective study that identified the serum level ≥ 1200 μg/mL as being associated with severe dengue was conducted in a paediatric population and used another definition for severe dengue [15]. So, a different cutoff value with greater sensitivity and specificity for severe dengue in a population of older adults should be evaluated, preferably in a prospective study.

We were unable to estimate a cut‐off of serum ferritin due to the low collection percentage in phases 1 (23.9%) and 2 (50%), which could have predicted progression to severe dengue. However, it is worth noting that serum ferritin levels were significantly higher in severe dengue cases in the critical and convalescence phases.

On the other hand, older age has consistently been identified as an important association with severe dengue and dengue‐related mortality. Epidemiological and clinical studies show that patients aged 60 years or older have higher rates of hospitalisation, organ dysfunction, intensive care unit admission, and death compared with younger adults. Age‐associated factors, including immunosenescence, chronic comorbidities and delayed clinical presentation, are considered contributors to poorer outcomes in this population [17].

Although most ferritin studies include mixed‐age populations, several investigations suggest an indirect link between advanced age, elevated ferritin levels and severe dengue outcomes. Observational studies have reported positive correlations between age and serum ferritin levels in dengue patients, along with longer hospital stays and higher complication rates. These findings suggest that a population of older adults may be more prone to exaggerated inflammatory responses, resulting in hyperferritinemia and increased disease severity [18]. Considering that the population is living longer due to increased life expectancy, studies on the population of older adults should be considered. There is a lack of studies evaluating prognostic factors for dengue in a population of older adults.

One of the few published studies is a large cohort focusing on a population of older adults diagnosed with dengue that demonstrates a high frequency of severe manifestations such as shock, acute kidney injury, liver dysfunction, and bleeding [17]. While ferritin was not the primary variable analysed in this study, the strong association between hyperferritinemia and severe dengue may support its relevance also in older adults. Given that ferritin levels tend to increase with age and comorbid inflammatory states, hyperferritinemia may have particular prognostic value in a population of older adult dengue patients [19].

Because ferritin testing is widely available and relatively inexpensive, it can represent a practical tool for risk stratification, especially in resource‐limited settings where dengue is endemic. Despite its potential utility, ferritin is a non‐specific marker and can be elevated in other infections and inflammatory conditions. Therefore, it should be interpreted in conjunction with clinical findings and other laboratory parameters, such as platelet count, haematocrit, liver enzymes and markers of inflammation [20]. Further prospective studies are needed to establish standardised ferritin cut‐off values and to validate its routine use in clinical practice.

Our study has limitations because it was retrospective and conducted in a single centre. Another limitation of the retrospective design was that the ferritin measurements were not standardised. For this reason, we obtained a few measurements of this biomarker at the earliest time of the disease, and it was not possible to establish a reliable prognostic value early for the outcomes evaluated. In addition, we must consider the limitation of classifying the period of illness (febrile, critical and convalescence) exclusively by day of illness, without individualised evaluation [11, 19]. In addition, alcohol consumption, obesity or iron profile were not evaluated, and these are factors known to influence serum ferritin levels [14].

Unfortunately, we were unable to assess the immunological status of patients in terms of primary versus post‐primary (or secondary) dengue, a distinction classically considered of major importance for the clinical evolution of dengue, due to the antibody‐dependent enhancement mechanism [16]. Although recent studies with robust methodology have also shown a high proportion of severe cases in children with primary dengue [13], we were unable to make this assessment due to the lack of quantitative serology or serial qualitative IgM and IgG evaluations during the course of the disease, which would also make this distinction possible, even if rapid diagnostic tests had been used for this purpose [21].

## 5 Conclusion

In this study of dengue virus infection in a population of older adults, serum ferritin levels were significantly increased in severe dengue, both in the critical and convalescence phases of disease, in comparison to non‐severe dengue. Although studies specifically designed to evaluate ferritin as a prognostic marker exclusively in a population of older adults dengue patients are limited, available data suggest that older adults with severe dengue frequently exhibit marked inflammatory responses consistent with elevated ferritin levels. Further age‐stratified and prospective studies are needed to define ferritin cut‐off values and clarify its role in risk stratification among a population of older adult patients.

## Conflicts of Interest

The authors declare no conflicts of interest.

## Data Availability

The data that support the findings of this study are available from the corresponding author upon reasonable request.
